# Metabolites with Insecticidal Activity from *Aspergillus fumigatus* JRJ111048 Isolated from Mangrove Plant *Acrostichum specioum* Endemic to Hainan Island

**DOI:** 10.3390/md15120381

**Published:** 2017-12-06

**Authors:** Zhikai Guo, Cuijuan Gai, Caihong Cai, Liangliang Chen, Shoubai Liu, Yanbo Zeng, Jingzhe Yuan, Wenli Mei, Haofu Dai

**Affiliations:** 1 Key Laboratory of Biology and Genetic Resources of Tropical Crops, Ministry of Agriculture, Institute of Tropical Bioscience and Biotechnology, Chinese Academy of Tropical Agricultural Sciences, Haikou 571101, China; guozhikai@itbb.org.cn (Z.G.); gaicuijuan@163.com (C.G.); caicaihong@itbb.org.cn (C.C.); chenliangliang502@163.com (L.C.); zhiwu19831113@163.com (S.L.); zengyanbo@itbb.org.cn (Y.Z.); yuanjingzhenpc@126.com (J.Y.); 2 Hainan Key Laboratory for Research and Development of Natural Products from Li Folk Medicine, Institute of Tropical Bioscience and Biotechnology, Chinese Academy of Tropical Agricultural Sciences, Haikou 571101, China

**Keywords:** *Acrostichum specioum*, mangrove endophyte, *Aspergillus fumigatus*, anhydride, fatty acid amide, insecticidal activity, antifungal activity

## Abstract

Fungi residing in mangroves are considered to be a bank of novel bioactive natural products. In the screening for bioactive metabolites from mangrove-derived fungi, the ethyl acetate extract of the fermentation broth of *Aspergillus fumigatus* JRJ111048, a fungus isolated from the leaves of the mangrove plant *Acrostichum specioum* endemic to Hainan island, was found to possess insecticidal activity against *Spodoptera litura*. Bioactivity-guided isolation lead to the discovery of seven metabolites **1**–**7**, including one new anhydride derivative aspergide (**1**), one new lipid amide 11-methyl-11-hydroxyldodecanoic acid amide (**2**), and five known compounds; α-ethyl glucoside (**3**), spiculisporic acid B (**4**), spiculisporic acid C (**5**), spiculisporic acid (**6**), and secospiculisporic acid B (**7**). Their structures were established by NMR spectroscopic and MS analyses, and by comparison of previously reported data. Insecticidal activity against *S. litura* and antifungal activity of these compounds were investigated. As a result, the new compound **1** showed potent insecticidal activity against newly hatched larvae of *S. litura*, and compound **4** displayed weak antifungal activity against *Candida albicans*.

## 1. Introduction

**Figure 1:**
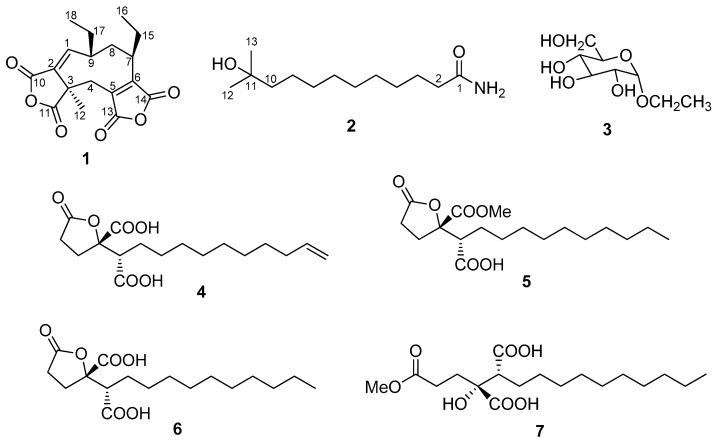
The chemical structures of compounds **1**–**7** isolated from *A. fumigatus* JRJ11104.

Marine-derived fungi, such as species of the genus *Aspergillus* (Trichocomaceae), are a prolific resource for discovering structurally diverse and biologically active secondary metabolites [1,2,3,4]. Owing to their unique ecological environments, mangrove plants, growing in the saline coastal habitats in the tropics and subtropics, are considered to be a promising source of novel endophytic fungi such as *Aspergillus* spp., which could produce versatile bioactive natural products with novel structures [5,6,7]. In our continuous search for new bioactive natural products from plant endophytic fungi [8,9,10,11,12], and as part of a program to investigate the chemical and biological diversity of the secondary metabolites from the endemic mangrove plants-associated fungi, we found a series of novel meroterpenes, sesquiterpenes, and glycoside and furan derivatives with diverse bioactivities [13,14,15,16]. During our ongoing investigation on bioactive secondary metabolites from mangrove-derived fungi, the ethyl acetate extract of the fermentation broth of *Aspergillus fumigatus* JRJ111048, a fungus isolated from the leaves of the mangrove plant *Acrostichum specioum* endemic to Hainan island, was found to possess insecticidal activity against *Spodoptera litura*. Further isolation by bioactivity-guided fractionation led to the discovery of seven metabolites (**1**–**7**), including one new anhydride derivative aspergide (**1**), one new fatty acid amide 11-methyl-11-hydroxyldodecanoic acid amide (**2**), and five known compounds; α-ethyl glucoside (**3**) [17], spiculisporic acid B (**4**) [18], spiculisporic acid C (**5**) [18], spiculisporic acid (**6**) [19], and secospiculisporic acid B (**7**) [20] (Figure 1), the structures of which were identified by NMR spectroscopic and MS analyses, as well as by comparison of previously reported data in the literature. In this paper, we report the isolation and structure elucidation of two new compounds, (**1**) and (**2**), as well as the insecticidal and antifungal activity of these isolated compounds.

## 2. Results

**Figure 2:**
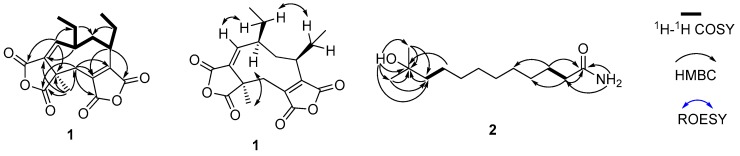
The key two-dimensional (2D) NMR correlations of **1** and **2**.

Aspergide (**1**) was isolated as a white powder. The molecular formula of **1** was determined as C_18_H_20_O_6_ (pointing to nine degrees of unsaturation) on the basis of the HR-ESI-MS, giving an [M − H]^−^ ion at *m*/*z* 331.11842, and the ^1^H and ^13^C NMR data (Table 1). The ^1^H NMR spectrum of **1** in CDCl_3_ showed the presence of three methyl groups (δ_H_ 0.81, 1.07, and 1.49), one olefinic proton (δ_H_ 7.00), four methylene groups (δ_H_ 3.28 and 2.66; 2.87 and 1.98; 1.86 and 1.14; and 1.67 and 1.50), and two aliphatic methine protons (δ_H_ 2.12 and 2.10). The ^13^C NMR and DEPT135 spectra of **1** showed the presence of 18 carbon resonances, including four carbonyl carbons (δ_C_ 173.7, 165.3, 164.5, and 163.7), four aromatic or double-bond carbons (δ_C_ 150.1, 148.4, 140.5, and 131.9), one quaternary carbon (δ_C_ 48.6), three methyl carbons (δ_C_ 12.4, 12.8, and 20.3), four aliphatic methylene carbons (δ_C_ 31.7, 28.4, 26.0, and 21.4), and two aliphatic methine carbons (δ_C_ 48.1 and 43.9). The complete assignments for **1** were deduced by detailed analyses of HSQC, ^1^H-^1^H COSY, HMBC, and ROESY (in Supplementary Materials). The ^1^H-^1^H COSY correlations of H-1 (δ_H_ 7.00)/H-9 (δ_H_ 2.10)/H_2_-8 (δ_H_ 2.87 and 1.98); of H-9/H_2_-17 (δ_H_ 1.67 and 1.50)/H_3_-18 (δ_H_ 0.81); of H_2_-8/H-7 (δ_H_ 2.12); and of H-7/H_2_-15 (δ_H_ 1.86 and 1.14)/H_3_-16 (δ_H_ 1.07) revealed a spin system, as shown in Figure 2. The HMBC correlations from H-1 to C-17, C-3, and C-10; from H_3_-12 (δ_H_ 1.49) to C-2, C-3, C-4 and C-11; from H-7 to C5 and C-14; and from H_2_-4 (δ_H_ 3.28 and 2.66) to C-5, C-6, C-11, and C-13 suggested the presence of a nine-membered ring, and revealed the positions of four carbonyl groups adjacent to C-2, C-3, C-5, and C-6. The relatively upfield chemical shifts of the four carbonyl carbons, in combination with the fact that only seven of the nine degrees of unsaturation were accounted for, supported the connectivity of C-10 to C-11 and of C-13 to C-14 through the remaining two oxygen atoms, forming two anhydride rings. The relative configuration of **1** was determined by inspection of the ROESY spectrum, which showed the correlations of H-9 with H_3_-12 and of H_2_-15 with H_2_-17, indicating that these two ethyl groups on C-7 and C-9 have same orientation, while the methyl group CH_3_-12 was on the other plane. However, the absolute stereochemistry of **1** has not been determined yet. Therefore, the structure of **1**, designated as aspergide, was elucidated as shown in Figure 1.

The molecular formula of 11-methyl-11-hydroxyldodecanoic acid amide (**2**) was found to be C_13_H_27_NO_2_ by HR-ESI-MS ([M + H]^+^ at *m*/*z* 230.2113), which, in combination with the ^1^H and ^13^C NMR data (Table 1), dictated one unsaturation equivalent. Analysis of the ^1^H, ^13^C, and HSQC spectra of **2** revealed the presence of two methyl groups [CH_3_-12 and CH_3_-13 (δ_H_ 1.05; δ_C_ 29.8)], nine aliphatic methylene groups (δ_H_ 1.20–2.02; δ_C_ 24.3–44.2), three exchangeable protons, one carbonyl group (δ_C_ 174.8), and one oxygenated quaternary carbon (δ_C_ 69.2). The planar structure of **2** was elucidated by comprehensive analyses of its two-dimensional (2D) NMR data (Figure 2), revealing a structure containing a long alkyl chain. Two terminal methyl groups were deduced by the HMBC correlations from H_3_-12 and H_3_-13 to C-10 (δ_C_ 44.2) and C-11 (δ_C_ 69.2). The exchangeable proton of 11-OH (δ_H_ 4.04) was assigned to C-11 through interpretation of the key HMBC correlations from 11-OH to C-10, C-11, C-12, and C-13. In addition, HMBC correlations from 1-NH_2_ (δ_H_ 7.20, 6.66) to the amide carbonyl carbon C-1 (δ_C_ 174.8) and C-2 (δ_C_ 35.6) established the amino group at the C-1 position. The alkyl chain composed of overlapping methylene protons was deduced from the COSY correlations among the methylene protons along with the clear HMBC correlations from H_2_-2 (δ_H_ 2.02) to C-1 and C-4 and from H_2_-3 (δ_H_ 1.47) to C-1 and C-5. Thus, the planar structure of **2** was identified as 11-methyl-11-hydroxyldodecanoic acid amide (Figure 1).

Compounds **1**–**7** were assessed in in vivo assays against newly hatched larvae of *S. litura* by mixing the sample into an artificial diet. As a result, compound **1** displayed potent insecticidal activity against newly hatched larvae of *S. litura* at the concentration of 20 µg/mL, and caused obvious effects such as reduced larval growth when compared with the untreated controls. Also, the pupae weight was reduced and the morphology of the adult was affected. At the larval stage, the treated groups showed mortality of 40.00 ± 10.00%, 46.67 ± 5.77%, 60.00 ± 0.00%, and 76.67 ± 5.77%, while the positive controls (azadirachtin) showed mortality of 86.67 ± 5.77%, 93.33 ± 5.77%, 100.00 ± 0.00%, and 100.00 ± 0.00% at 7th, 10th, 14th, and 20th days, respectively (Table 2). The antifungal activities of these compounds were also tested in vitro towards *Colletotrichum gloeosporioides*, *Fusarium oxysporum* f. sp. cubense race 4, and *C. albicans* ATCC10231 by the filter paper disc diffusion method. In the assays, only compound **4** showed weak antifungal effects on *C. albicans* with diameters of inhibition zones of 8.6 mm at the concentration of 20 mg/mL, as compared to the positive control ketoconazole with an inhibition diameter of 29.2 mm.

## 3. Materials and Methods

### 3.1. General Experimental Procedures

Optical rotations were measured in MeOH on a Rudolph Autopol III automatic polarimeter. The UV spectrum was recorded on a Shimadzu UV-2550 spectrophotometer. The IR spectra were obtained on a Nicolet 380 FT-IR instrument. The HR-ESI-MS spectra were acquired using an Agilent 1260 Infinity liquid chromatography coupled to a 6230 time of flight. One-dimensional (1D) and 2D NMR spectra were conducted on a Bruker AvanceIII 500 MHz NMR spectrometer at 500 MHz for ^1^H NMR and 125 MHz for ^13^C NMR or Bruker Avance III Ultrashield 700 MHz NMR spectrometer at 700 MHz for ^1^H NMR and 175 MHz for ^13^C NMR. The chemical shifts were given in δ (ppm) and referenced to the solvent signal (CDCl_3_, δ_H_ 7.26, δ_C_ 77.1; DMSO-*d*_6_, δ_H_ 2.50, δ_C_ 39.5). Column chromatography (CC) was performed on silica gel (200–300 mesh, Qingdao Marine Chemical Inc., Qingdao, China), Octadecylsilyl (40–70 µm, Fuji Silysia Chemical Ltd., Nagoya, Japan), and Sephadex LH-20 (GE Healthcare Bio-Sciences AB, Uppsala, Sweden). HPLC was carried out on a Varian semi-preparative HPLC system (Woburn, MA, USA) with an Alltima C18 column (250 × 10.0 mm, 5 µm, CH_3_OH-H_2_O, 2 mL/min) for purification. Thin-layer chromatography (TLC) was performed with silica gel GF254 (Marine Chemical Inc., Qingdao, China). All chemicals used in this study were of analytical grade and HPLC grade.

### 3.2. Fungal Material and Cultivation

The fungus *A. fumigatus* (JRJ111048) was isolated by one of the authors (Z.G.) from a healthy leaf of endemic mangrove plant *A. specioum*, collected from the natural mangrove forest of Dongzhaigang in Haikou, Hainan Province, China, in October 2013, which was identified by Dr. Shoubai Liu, School of the Tropical Agriculture and Foresty, Hainan University. The isolate was identified as *A. fumigatus* on the base of its morphological characteristics and partial 18S rDNA gene sequence data (GenBank accession number MF817615) by a National Center for Biotechnology Information Basic Local Alignment Search Tool search. A voucher specimen, ITBBF14155, was deposited at the Key Laboratory of Biology and Genetic Resources of Tropical Crops, Ministry of Agriculture, Institute of Tropical Bioscience and Biotechnology, Chinese Academy of Tropical Agriculture Sciences, Haikou, China. The strain was grown on a Potato Dextrose Agar medium plate at 28 °C for 5 days. Then six small agar plugs (0.5 × 0.5 cm^2^) with mycelia were inoculated into each 1-L Erlenmeyer flask, each containing 300 mL Malt Extract liquid media (consisting of 10 g/L malt extract, 20 g/L sucrose, 2 g/L peptone, and deionized water). Fermentation was conducted on a rotary shaker with 180 rpm at 28 °C for 12 days.

### 3.3. Extraction and Isolation

The filtrate of fermented broth (35 L) was harvested and extracted four times with ethyl acetate at room temperature. The organic solvent was removed under reduced pressure to afford a crude extract (6.8 g). Then the extract was fractionated by silica gel (200–300 mesh) CC eluting with a gradient of CHCl_3_-MeOH (*v*/*v*, 100:0, 100:1, 100:2, 100:4, 100:8, 100:16, 100:32, 0:100) to give eight fractions (Fr.1–8). Fraction 2 (432 mg) was subjected to ODS CC with MeOH-H_2_O (*v*/*v*, 30:70, 40:60, 50:50, 60:40, 70:30, 80:20, 100:0) to give seven subfractions (Fr.2.1–Fr.2.7), and then Fr.2.4 (62 mg) was further purified by Sephadex LH-20 CC using MeOH as an eluent to afford compound **1** (7.6 mg). Fraction 4 (360 mg) was separated by Sephadex LH-20 CC eluting with MeOH to give five subfractions (Fr.4.1–Fr.4.5), and Fr.4.3 (55 mg) was further purified by semipreparative HPLC (CH_3_CN-H_2_O, 75%) to yield **2** (6.2 mg). Fraction 5 (753 mg) was isolated by ODS CC using MeOH-H_2_O (*v*/*v*, 30:70, 40:60, 50:50, 60:40, 70:30, 80:20, 100:0) as an eluent to give seven subfractions (Fr.5.1–Fr.5.7), and purification of the resulting Fr.5.6 (226 mg) by semipreparative HPLC (CH_3_OH-H_2_O, 70%) afforded **4** (11.5 mg), **5** (9.3 mg), **6** (18.7 mg), and **7** (21.2 mg). Fraction 5.4 (73 mg) was purified by Sephadex LH-20 CC, and eluted with MeOH to produce **3** (6.2 mg).

Aspergide (**1**): white powder; ${\left[\alpha \right]}_{D}^{25}$ +290 (*c* 0.05, MeOH); IR (neat) ν_max_ 3444, 2080, 1633, 1017, 555 cm^−1^; UV (MeOH) λ_max_ (log ε) 216 (4.05), 261 (3.45) nm; ^1^H and ^13^C NMR data see Table 1; HR-ESI-MS *m*/*z* 331.11842 [M − H]^−^ (calcd. for C_18_H_19_O_6_, 331.1182).

*11-Methyl-11-hydroxyldodecanoic acid amide* (**2**): white powder; IR (neat) ν_max_ 3450, 3342, 3180, 2933, 2855, 1742, 1433, 1061, 651 cm^−1^; ^1^H and ^13^C NMR data see Table 1; HR-ESI-MS *m*/*z* 230.2113 [M + H]^+^ (calcd. for C_13_H_28_NO_2_, 230.2115).

### 3.4. Antifungal Activity Assay

The antifungal activities of these isolated compounds were evaluated against *Colletotrichum gloeosporioides*, *Fusarium oxysporum* f. sp. cubense race 4, and *C. albicans* ATCC10231 by using the agar diffusion method with 6-mm filter paper discs at the concentration of 20 mg/mL in accordance with the previous report [11], and ketoconazole was used as the positive control.

### 3.5. Insecticidal Activity Assay

*S. litura* eggs were collected from the leaves of lotus (*Nelumbo nucifera* G.) in Guilinyang, Meilan Disctrict, Haikou, P.R. China, where pesticides had not been applied. Then these eggs were fed daily with meridic diet until they reproduced more generations under 25 ± 1 °C and a relative humidity of 80%. The insecticidal activity was investigated by adding sample to the diet of the newly hatched larvae. In the test, there were three groups, each containing 10 larvae. The tested compounds were dissolved in acetone at the concentration of 1 mg/mL. Meridic diet was pipetted into 12-well plates (1 mL/well), and then the tested compounds (20 µL/well) were added when they were solidified. Newly hatched larvae were raised under 25 ± 1 °C and a relative humidity of 80%. In the tests, Azadirachtin was used as the positive control, while acetone was used as the blank control. The number of dead larvae was recorded on the 7th, 10th, 14th, and 20th day after treatment, respectively, and then the mortality was calculated. The corrected mortality (CM) was calculated by the following index:

CM = (T − C) × 100/(1 − C)(1)

where T is the mortality percentage of the tested compounds, and C is the mortality percentage in the blank control.

## 4. Conclusions

In summary, one new anhydride derivative aspergide (**1**), one new lipid amide 11-methyl-11-hydroxyldodecanoic acid amide (**2**), along with five known compounds; α-ethyl glucoside (**3**), spiculisporic acid B (**4**), spiculisporic acid C (**5**), spiculisporic acid (**6**), and secospiculisporic acid B (**7**), were isolated from the ethyl acetate extract of the fermentation broth of *A. fumigatus* JRJ111048, isolated from the leaves of *A. specioum*, a mangrove plant endemic to Hainan island. Their chemical structures were established by NMR and MS analyses, and by comparison of previously reported data. Compound **4** displayed weak antifungal activity against *C. albicans*. The new compound **1** showed potent insecticidal activity against newly hatched larvae of *S. litura*, indicating that it could be used as a lead compound in insect-control agents or for managing the field population of *S. litura* pests in agriculture. These results indicate that mangrove-derived fungi are a prolific resource of new bioactive natural products.

## Figures and Tables

**Table 1:** ^1^H and ^13^C NMR spectroscopic data for compounds **1** and **2**.

No.	1 ^a^		2 ^b^	
	δ_H_, mult. (*J* in Hz)	δ_C_	δ_H_, mult. (*J* in Hz)	δ_C_
**1**	7.00, d (12.5)	150.1, CH		174.8, C
1-NH_2_			7.20, br s, 6.66, br s	
**2**		131.9, C	2.02, t (7.7)	35.6, CH_2_
**3**		48.6, C	1.47, m	25.5, CH_2_
**4**	3.28, d (13.0), 2.66, d (13.0)	31.7, CH_2_	1.20–1.27, m ^c^	29.2 ^d^, CH_2_
**5**		140.5, C	1.20–1.27, m ^c^	29.3 ^d^, CH_2_
**6**		148.4, C	1.20–1.27, m ^c^	29.4 ^d^, CH_2_
**7**	2.12, m, overlap	48.1, CH	1.20–1.27, m ^c^	29.6 ^d^, CH_2_
**8**	2.87, m, 1.98, m	28.4, CH_2_	1.20–1.27, m ^c^	30.3 ^d^, CH_2_
**9**	2.10, m, overlap	43.9, CH	1.28, m	24.3, CH_2_
**10**		163.7, C	1.31, m	44.2, CH_2_
**11**		173.7, C		69.2, C
11-OH			4.04, s	
**12**	1.49, s	20.3, CH_3_	1.05, s	29.8, CH_3_
**13**		165.3, C	1.05, s	29.8, CH_3_
**14**		164.5, C		
**15**	1.86, m, 1.14, m	21.4, CH_2_		
**16**	1.07 t (7.0)	12.4, CH_3_		
**17**	1.67, m, 1.50, m	26.0, CH_2_		
**18**	0.81, t (7.5)	12.8, CH_3_		

^a^ Acquired in CDCl_3_; ^b^ Acquired in DMSO-*d*_6_; ^c^ overlapping signals; ^d^ Interchangeable signals.

**Table 2:** Insecticidal activity of compound **1**.

Samples	Mortality Rate (%)			
	7 d	10 d	14 d	20 d
Compound **1**	40.00 ± 10.00	46.67 ± 5.77	60.00 ± 0.00	76.67 ± 5.77
Azadirachtin	86.67 ± 5.77	93.33 ± 5.77	100.00 ± 0.00	100.00 ± 0.00
Control	0.00 ± 0.00	3.33 ± 5.77	3.33 ± 5.77	6.67 ± 5.77

## Supplementary Materials

The NMR and MS spectra for **1** and **2** are available online at www.mdpi.com/1660-3397/15/12/381/s1.
