# Supplementary material for: Metabolites with Insecticidal Activity from Aspergillus fumigatus JRJ111048 Isolated from Mangrove Plant Acrostichum specioum Endemic to Hainan Island
Source: Mar Drugs. 2017 Dec 6;15(12):381. doi: 10.3390/md15120381 (PMC5742841; doi:10.3390/md15120381)

Supporting Information

Metabolites with Insecticidal activity from *Aspergillus fumigatus* JRJ111048 Isolated from Mangrove Plant *Acrostichum specioum* Endemic to Hainan Island

Zhikai Guo‡, Cuijuan Gai‡, Caihong Cai, Liangliang Chen, Shoubai Liu, Yanbo Zeng, Jingzhe Yuan, Wenli Mei* and Haofu Dai*

Key Laboratory of Biology and Genetic Resources of Tropical Crops, Ministry of Agriculture, Institute of Tropical Bioscience and Biotechnology, Chinese Academy of Tropical Agricultural Sciences, and Hainan Key Laboratory for Research and Development of Natural Products from Li Folk Medicine, Haikou 571101, Hainan, People's Republic of China; guozhikai@itbb.org.cn (Z.G.); gaicuijuan@163.com (C.G.); caicaihong@itbb.org.cn (C.C.); 452184570@qq.com (L.C.); zhiwu19831113@163.com (S.L.); zengyanbo@itbb.org.cn (Y.Z.); yuanjingzhenpc@126.com (J.Y.)

* Correspondence: meiwenli@itbb.org.cn (W.M.); daihaofu@itbb.org.cn (H.D.)

‡ These authors contributed equally to this work.

Figure S1. ^{1}H NMR (500 MHz, $CDCl_3$) spectrum of compound **1**

Figure S2. ^{13}C NMR (125 MHz, $CDCl_3$) spectrum of compound **1**

Figure S3. DEPT135 spectrum of compound **1** in $CDCl_3$

Figure S4. HSQC spectrum of compound **1** in $CDCl_3$

Figure S5. ^{1}H-^{1}H COSY spectrum of compound **1** in $CDCl_3$

Figure S6. HMBC spectrum of compound **1** in $CDCl_3$

Figure S7. ROESY spectrum of compound **1** in $CDCl_3$

Figure S8. Enlarged ROESY spectrum (δ_H 0.5-4.0 ppm) of compound **1** in $CDCl_3$

Figure S9. HR-ESI-MS spectrum of compound **1** in MeOH

Figure S10. ^{1}H NMR (700 MHz, DMSO-d_6) spectrum of compound **2**

Figure S11. ^{13}C NMR (175 MHz, DMSO-d_6) spectrum of compound **2**

Figure S12. DEPT135 spectrum of compound **2** in DMSO-d_6

Figure S13. HSQC spectrum of compound **2** in DMSO-d_6

Figure S14. ^{1}H-^{1}H COSY spectrum of compound **2** in DMSO-d_6

Figure S15. HMBC spectrum of compound **2** in DMSO-d_6

Figure S16. HR-ESI-MS spectrum of compound **2** in MeOH

Figure S1. ^{1}H NMR (500 MHz, $CDCl_3$) spectrum of compound **1**

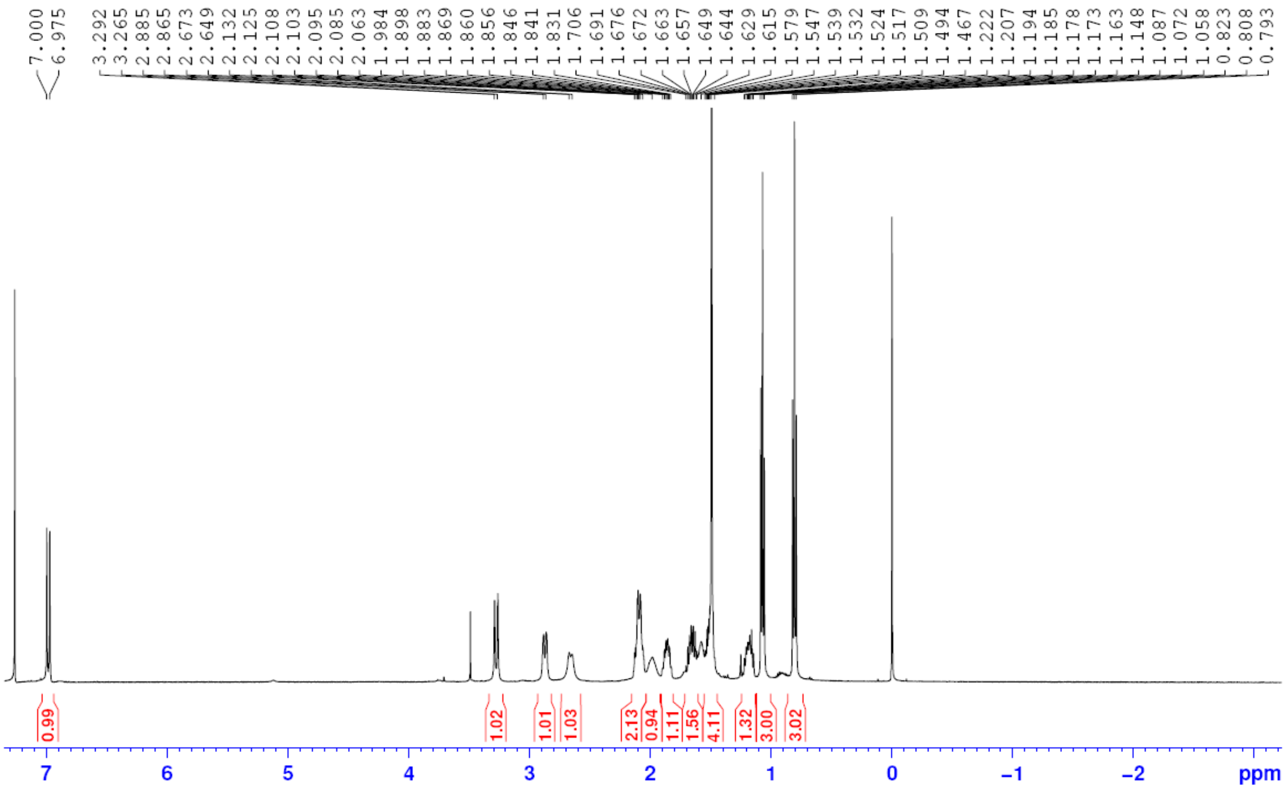

Figure S2. ^{13}C NMR (125 MHz, $CDCl_3$) spectrum of compound **1**

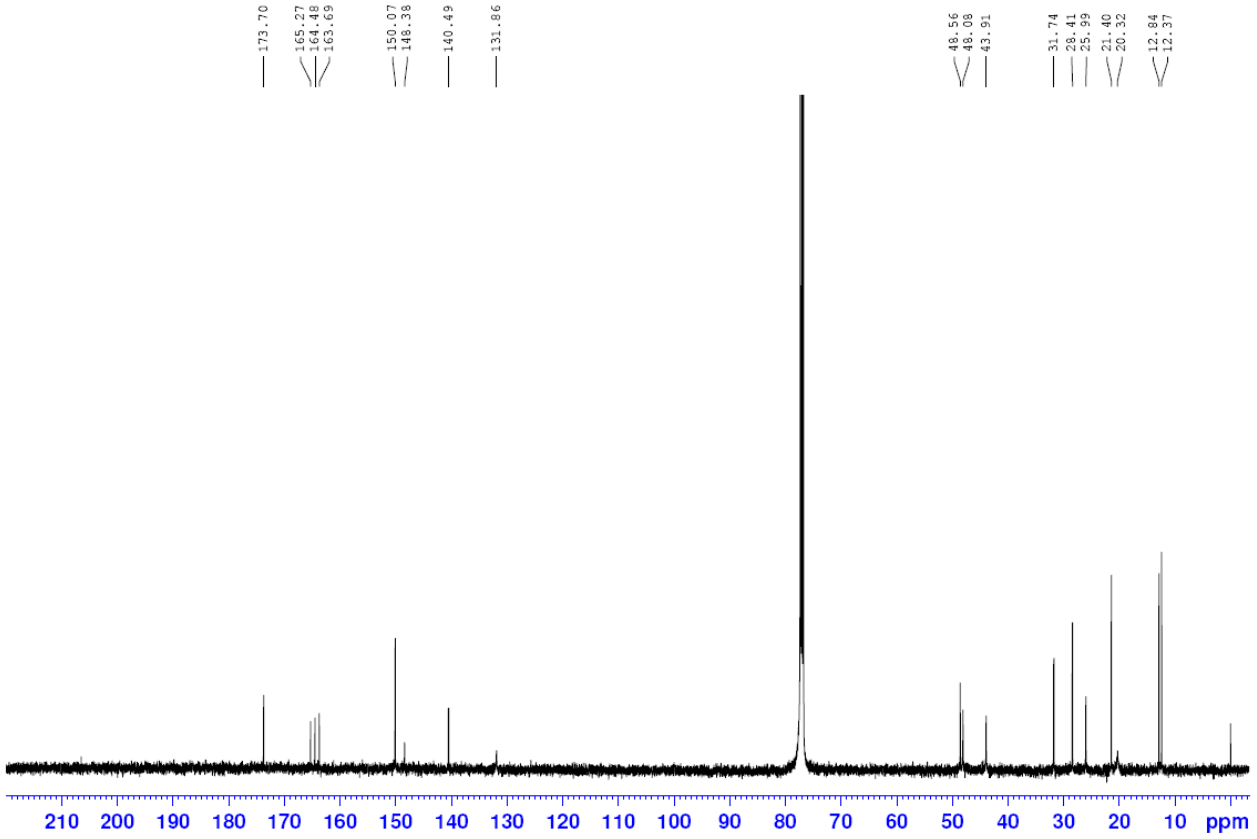

Figure S3. DEPT135 spectrum of compound **1** in $CDCl_3$

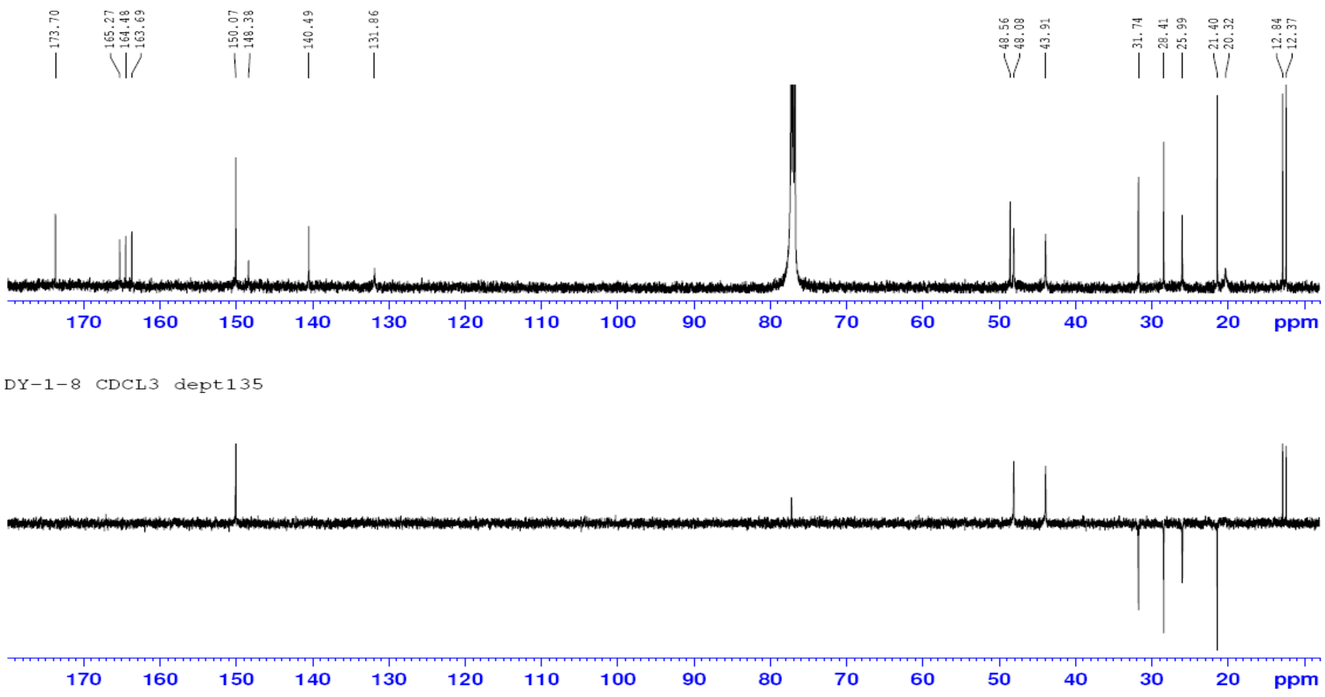

Figure S4. HSQC spectrum of compound **1** in $CDCl_3$

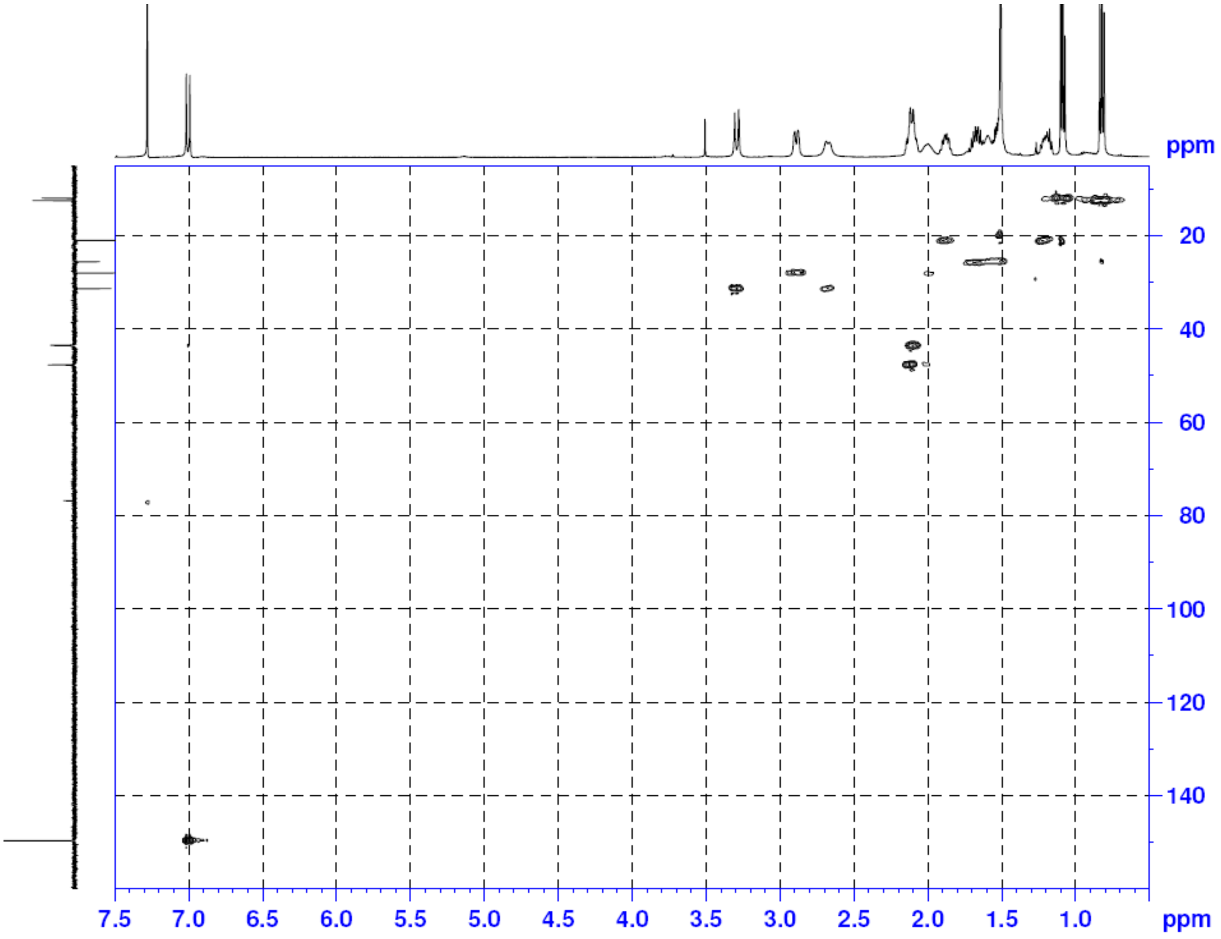

Figure S5. ^{1}H-^{1}H COSY spectrum of compound **1** in $CDCl_3$

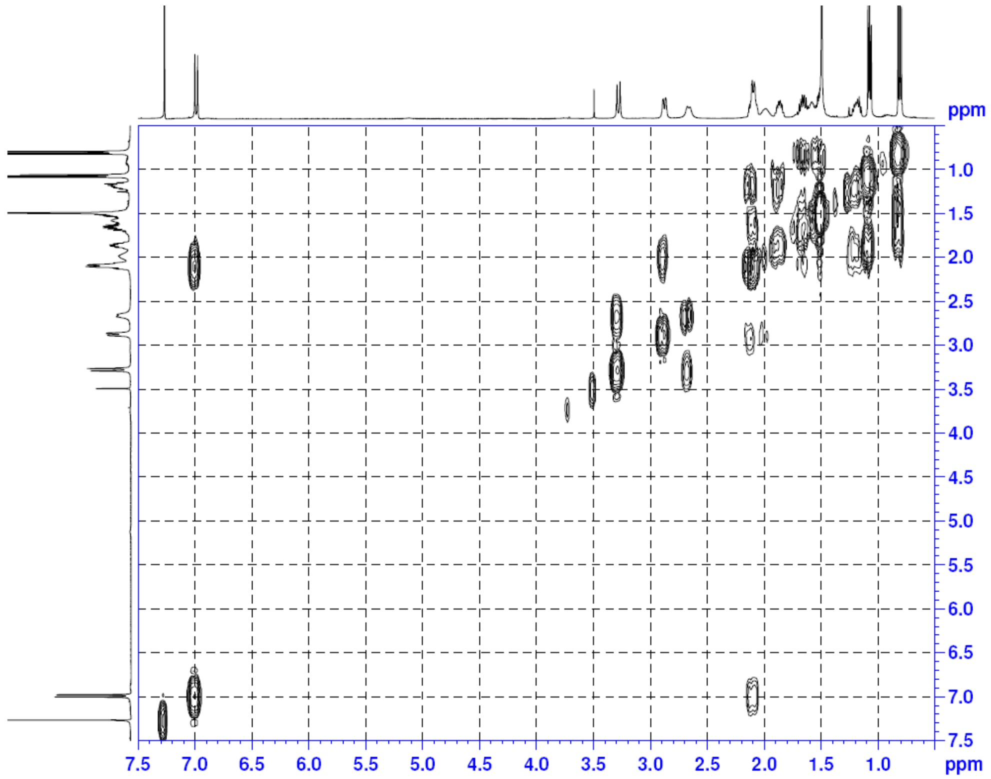

Figure S6. HMBC spectrum of compound **1** in $CDCl_3$

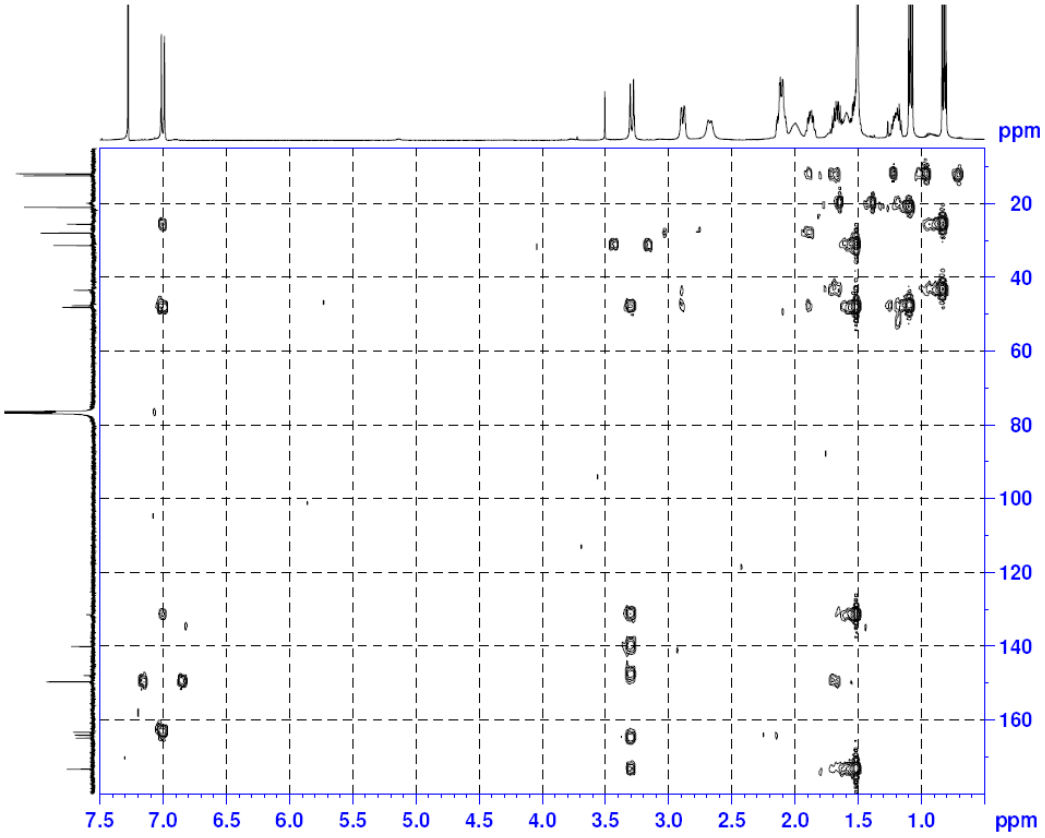

Figure S7. ROESY spectrum of compound **1** in $CDCl_3$

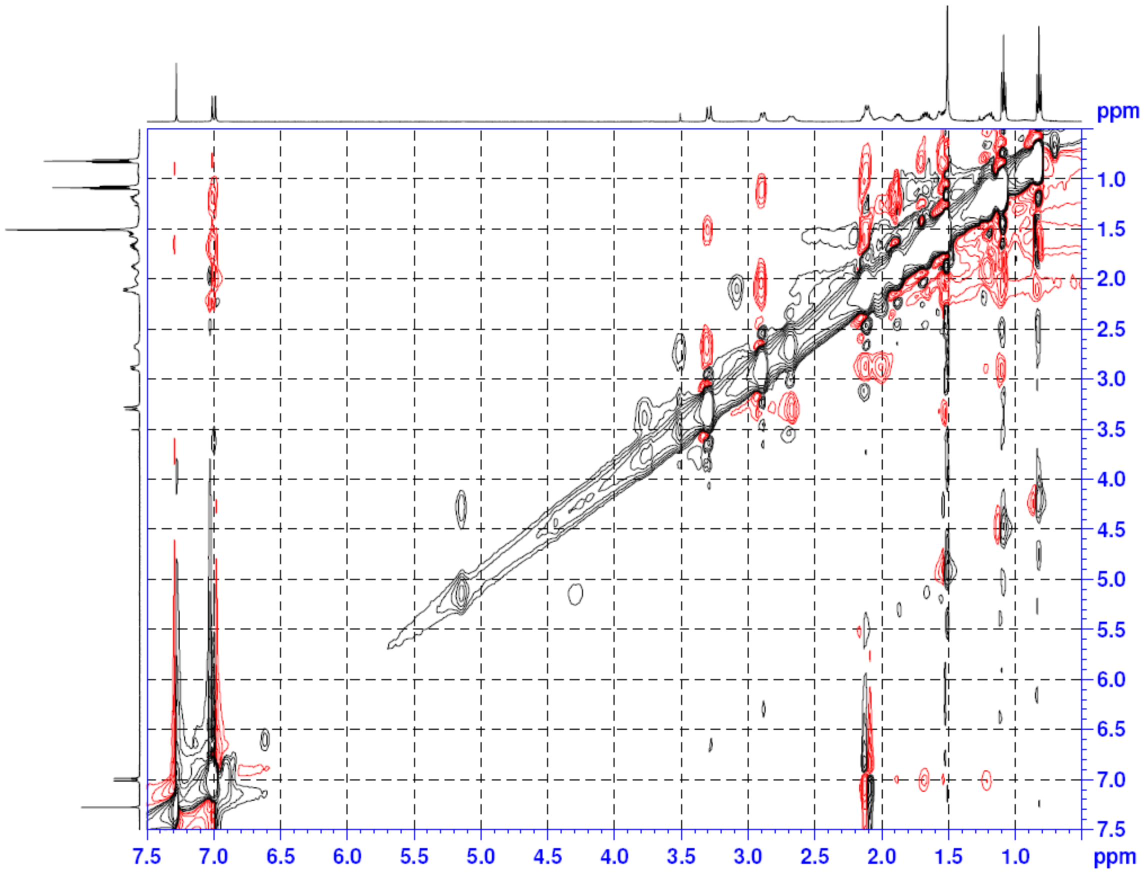

Figure S8. Enlarged ROESY spectrum (δ_H 0.5-4.0 ppm) of compound **1** in $CDCl_3$

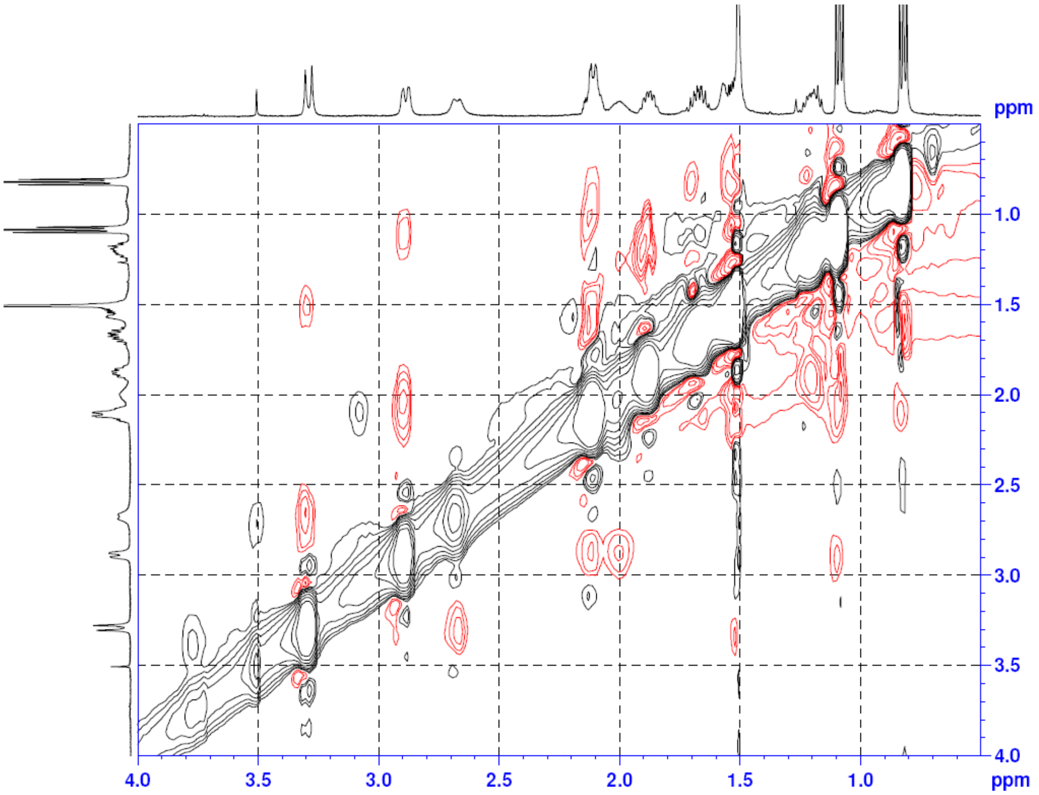

Figure S9. HR-ESI-MS spectrum of compound **1** in MeOH

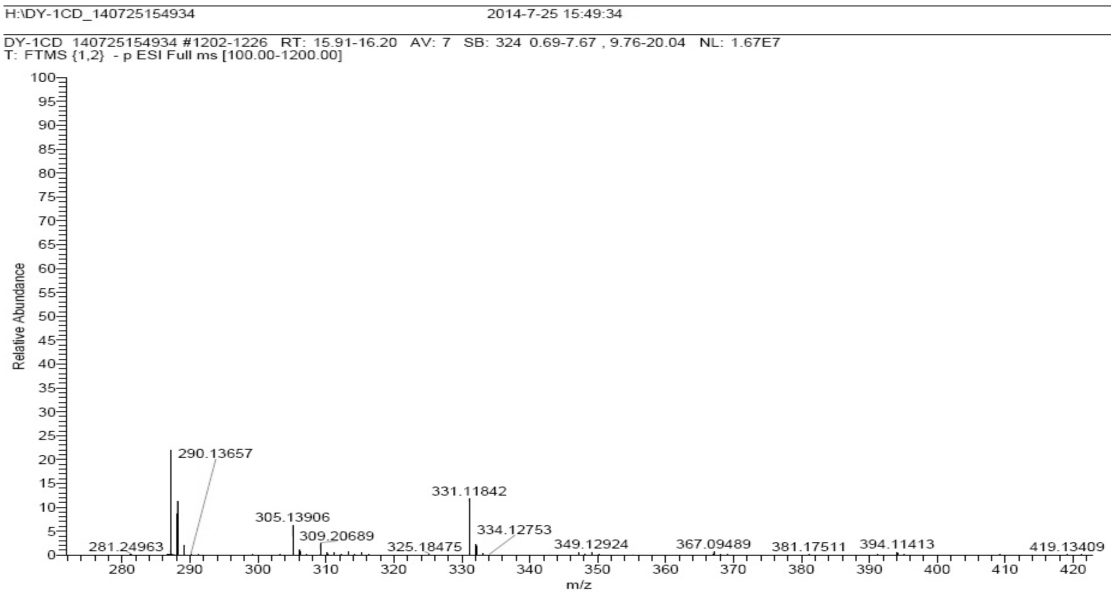

Figure S10. ^{1}H NMR (700 MHz, DMSO-d_6) spectrum of compound **2**

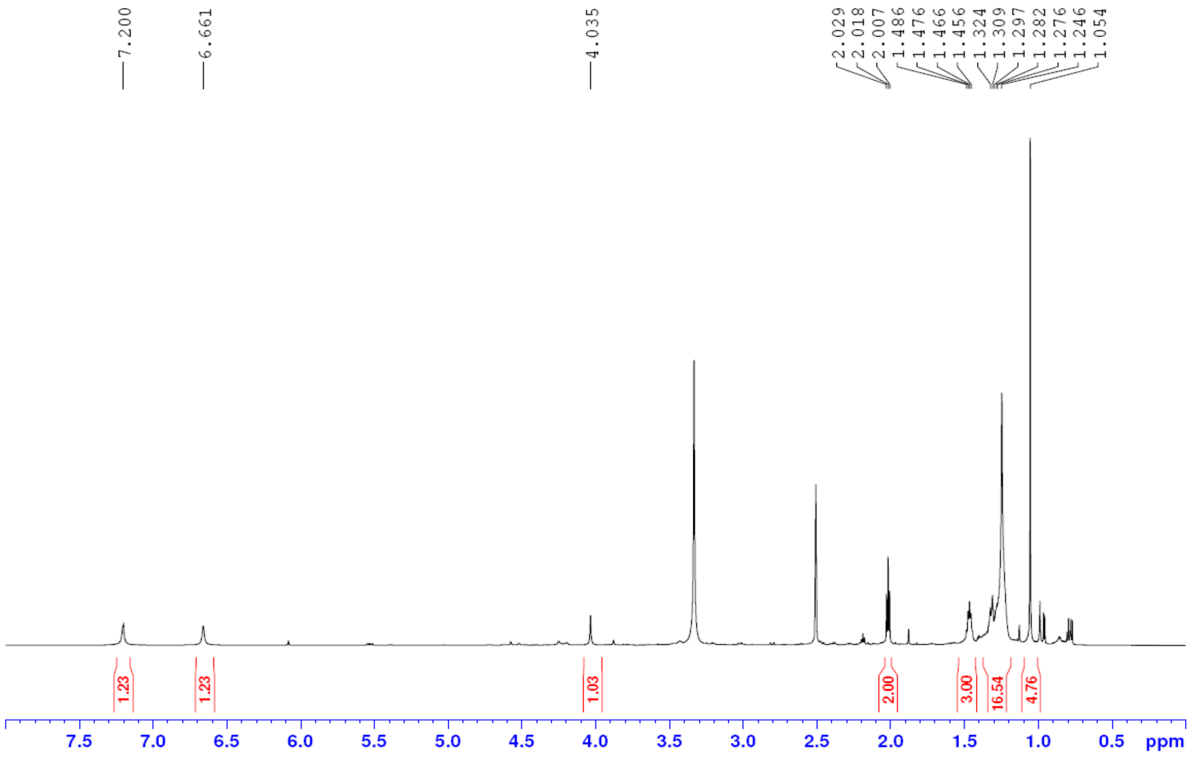

Figure S11. ^{13}C NMR (175 MHz, DMSO-d_6) spectrum of compound **2**

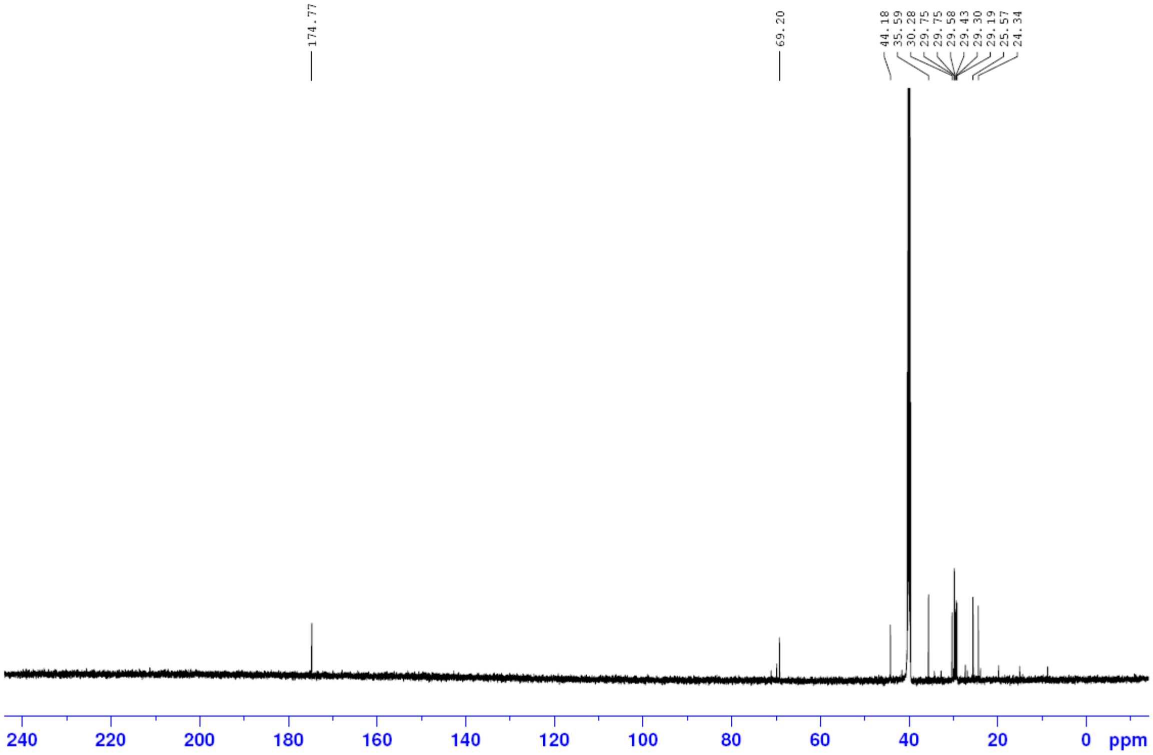

Figure S12. DEPT135 spectrum of compound **2** in DMSO-d_6

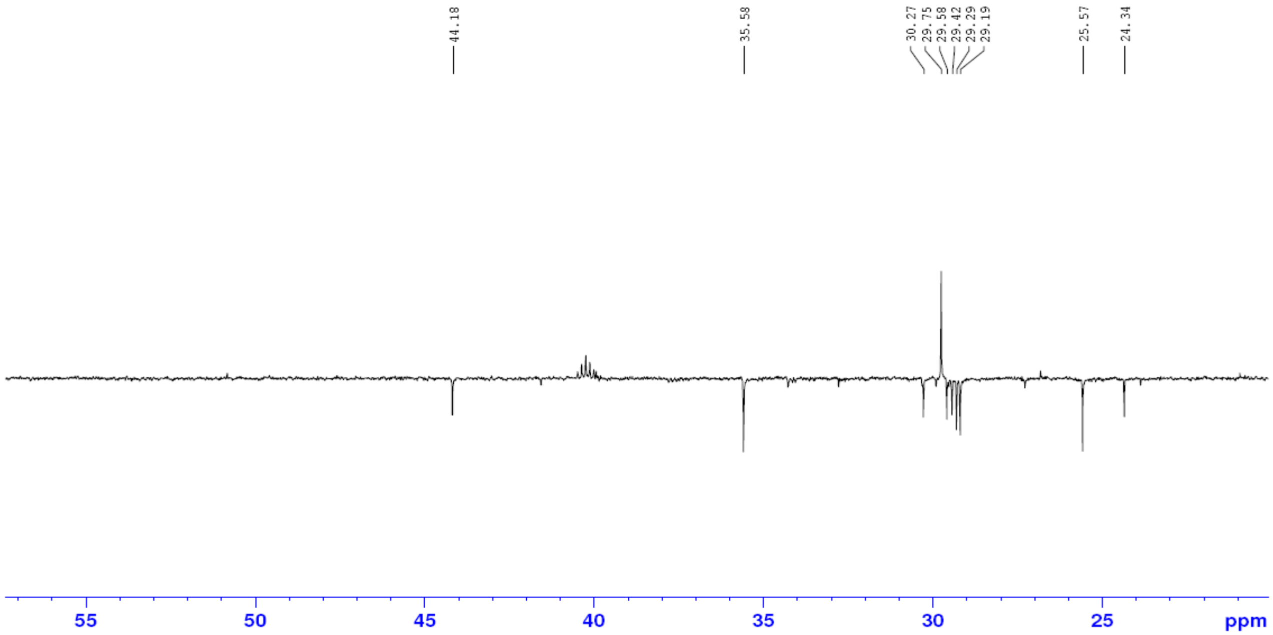

Figure S13. HSQC spectrum of compound **2** in DMSO-d_6

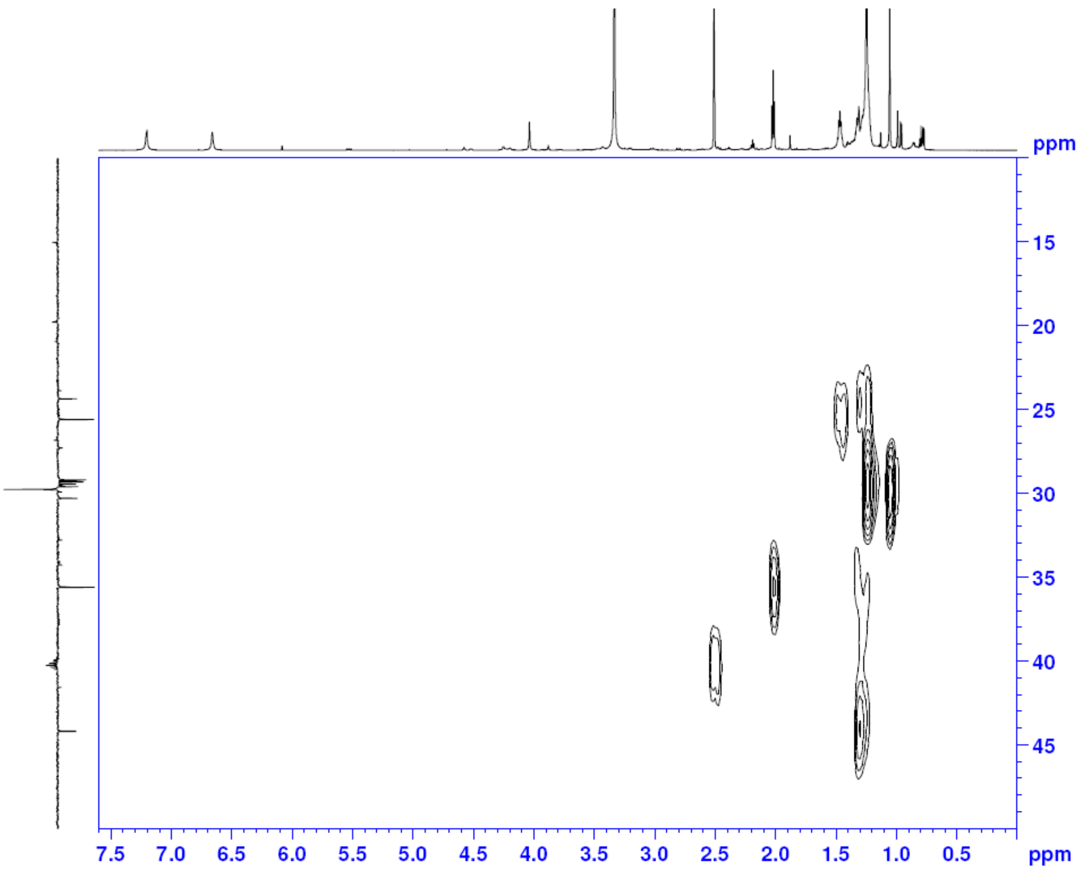

Figure S14. ^{1}H-^{1}H COSY spectrum of compound **2** in DMSO-d_6

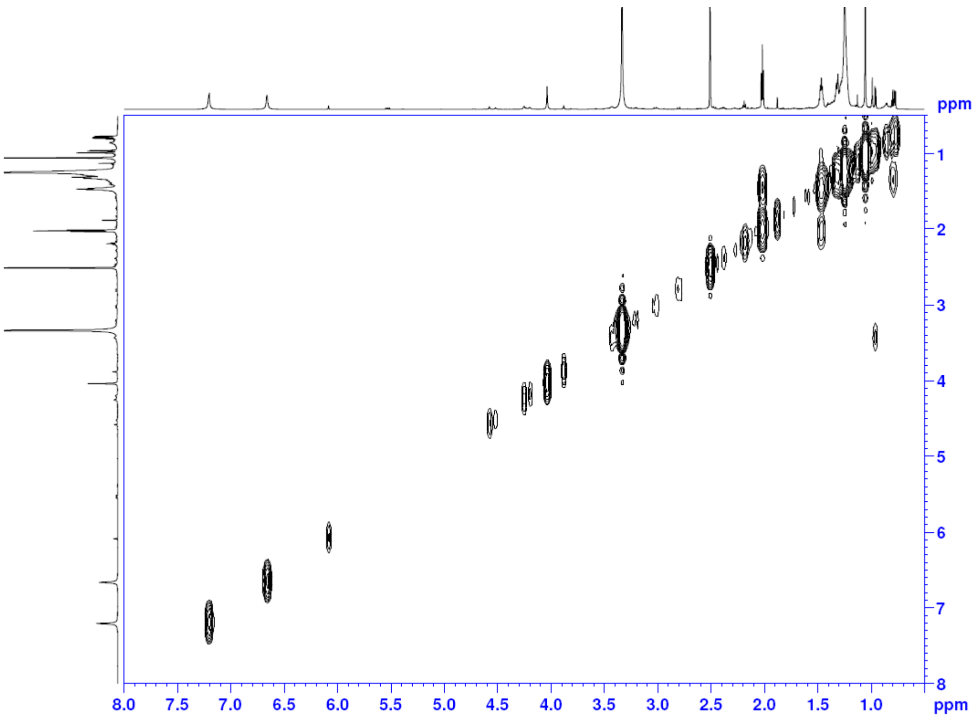

Figure S15. HMBC spectrum of compound **2** in DMSO-d_6

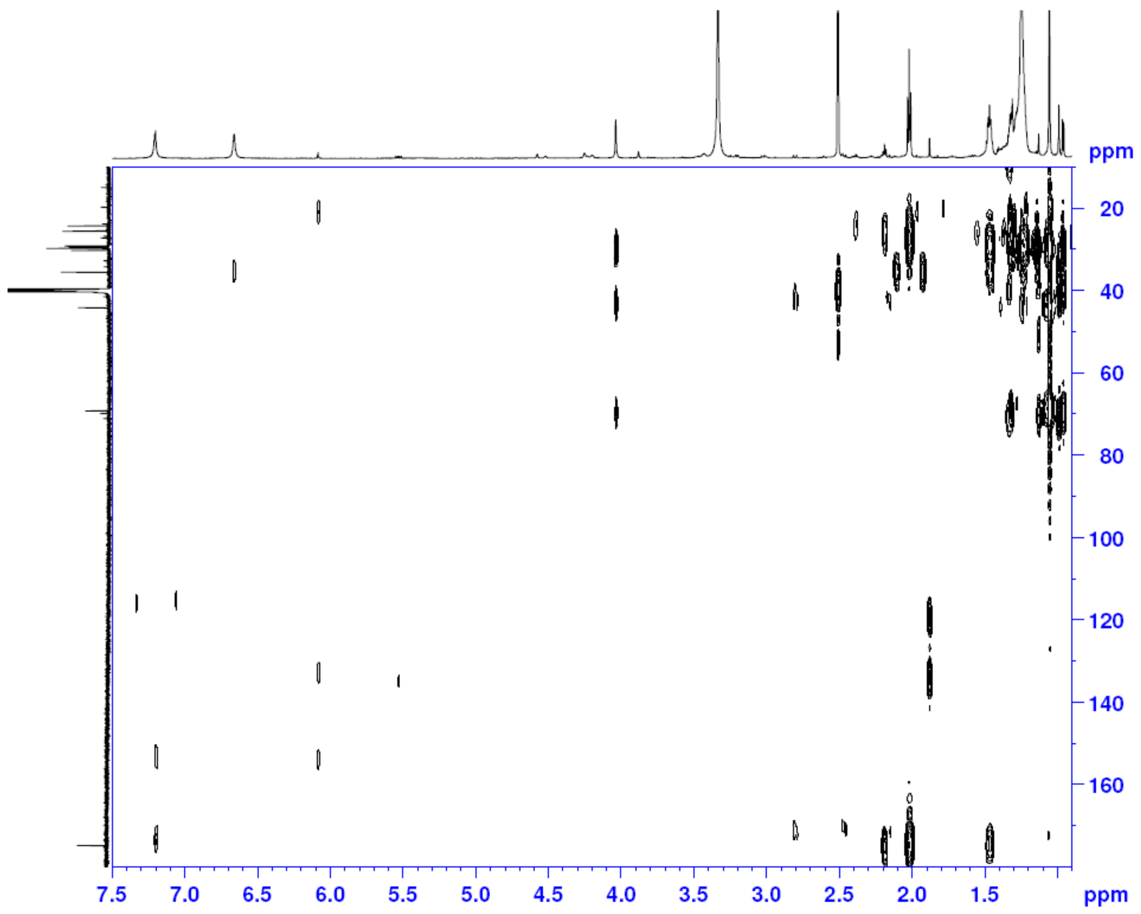

Figure S16. HR-ESI-MS spectrum of compound **2** in MeOH

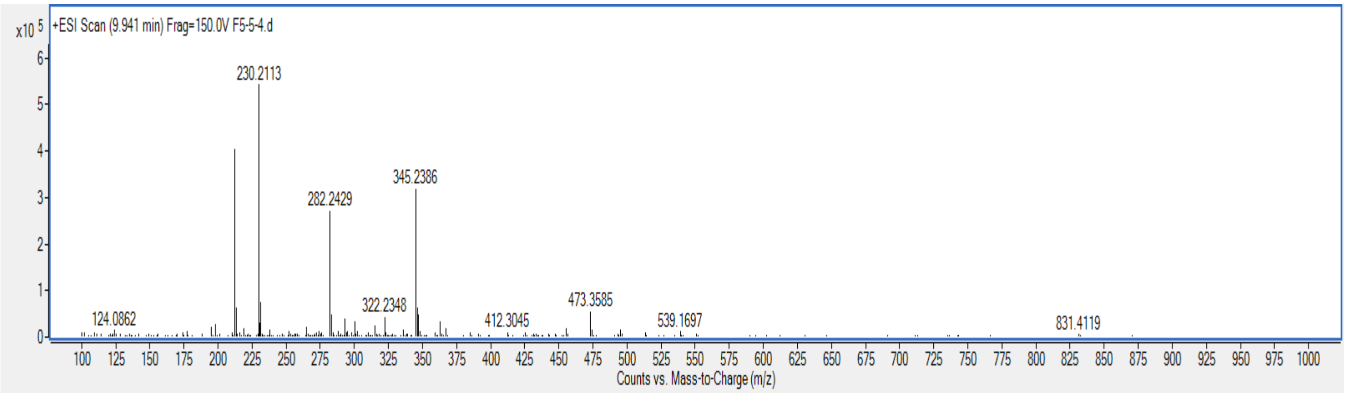

Supplement: Supplementary file 1 [file marinedrugs-15-00381-s001.pdf]
